# Good Potable Water

**Published:** 1874-04

**Authors:** 


### GOOD POTABLE WATER.

E. Reichart has contributed to the *Archiv der Pharmacie* an aiticle on the qualities peculiar to good potable water, and from this we extract the following interesting points:

Pure water is essential to health. The purity of water is to be determined by chemical analysis, and not as formerly by taste, smell, color, etc., or by general notions of physicians or others, depending on what is supposed to be the source of the water, or on the fancied effects of its use.

Generally it is necessary to determine only a few of the impurities of water to judge of its fitness for use. These impurities are Cl, $H_2SO_4$, CaO, MgO and, above all, $HNO_3$. Water is unfit for a beverage in proportion as these substances are present, and, as river water contains far more of them than spring water, it is in so far less suited for drinking. For this reason the Vienna Water Commission decided rightly that spring water is better for drinking than filtered river water. Cities that have constructed expensive apparatus for filtering river water will throw it aside hereafter, on sanitary grounds, and bring purer water from mountain springs, when possible, after the example of the old Romans.

